# Supplementary material for: Noblella thiuni sp. n., a new (singleton) species of minute terrestrial-breeding frog (Amphibia, Anura, Strabomantidae) from the montane forest of the Amazonian Andes of Puno, Peru
Source: PeerJ. 2019 Apr 23;7:e6780. doi: 10.7717/peerj.6780 (PMC6485238; doi:10.7717/peerj.6780)
Supplement: Appendix S1 — List of specimens of similar species examined for this work. Collection abbreviations: CORBIDI –Centro para Ornitología y Biodiversidad, Lima; KU –Kansas University, Lawrence; MHNC –Museo de Historia Natural de la Universidad San Antonio Abad, Cusco; MHNG–Muséum d’Histoire Naturelle, Geneva; MUSM –Museo de Historia Natural de la Universidad de San Marcos, Lima. [file peerj-07-6780-s001.docx]

**Appendix 1. List of specimens of similar species examined for this work.**

Collection abbreviations: CORBIDI –Centro para Ornitología y Biodiversidad, Lima; KU – Kansas University, Lawrence; MHNC – Museo de Historia Natural de la Universidad San Antonio Abad, Cusco; MHNG– Muséum d’Histoire Naturelle, Geneva; MUSM – Museo de Historia Natural de la Universidad de San Marcos, Lima.

*Noblella duellmani* (2 specimens): PERU: Pasco: Santa Barbara, KU 315004–05.

*Noblella heyeri* (3 specimens): PERU: Piura: 33 km SW Huancabamba, KU 196529 (holotype), 196530–31 (paratypes).

*Noblella lochites* (2 specimens): ECUADOR: Morona-Santiago: Río Piuntza, KU 147070 (holotype); ECUADOR: Pastaza: Mera, KU 177356.

*Noblella madreselva* (2 specimens): PERU: Cusco: Provincia La Convención, Madre Selva (Santa Ana), CORBIDI 15769 (holotype); CORBIDI 15770 (paratopotype).

*Noblella myrmecoides* (5 specimens): PERU: Loreto: lower Rio Napo region, E bank Rio Yanayacu, ca 90 km N Iquitos, KU 206120; Quebrada Oran, ca 5 km N Rio Amazonas, 85 km NE Iquitos, KU 206121; Quebrada Vasquez, N side of lower Rio Tahuayo, KU 220577, 220578, 220579.

*Noblella* cf. *myrmecoides* (23 specimens): PERU: Cusco: Provincia Paucartambo, Kosñipata, MHNG 2606.82–84, MUSM 21072–80, 30426–29, 30458–60; Madre de Dios: Provincia Manu, Los Amigos Conservation Concession, MUSM 27261, 24219, 24251, 24266, 27274−75.

*Noblella pygmaea* (15 specimens): PERU: Cusco: Provincia Paucartambo, Kosñipata, MHNG 2725.29–30, MUSM 24535–36, 26306–7, 26318–20, 30423–24, 30453–54, MTD 47286–87.

*Psychrophrynella bagrecito* (14 specimens): PERU: Cusco: Quispicanchis: Marcapata, Río Marcapata, below Marcapata, ca. 2740 m, KU 196512 (holotype), KU 196513–18, 196520–21, 196523–25 (all paratypes); La Convención: Hacienda Huyro between Huayopata and Quillabamba, 1830 m, KU 196527–28.

*Psychrophrynella chirihampatu* (27 specimens): PERU: Cusco: Provincia Paucartambo, Área de Conservación Privada (ACP) Ukumari Llaqta, Comunidad Campesina de Japu, 2730 – 3000 m, CORBIDI 16495–16499, CORBIDI 16501–16509, CORBIDI 16696, MHNC 14656, MHNC 14658, MHNC 14661–14662, MHNC 14664, MHNC 14666–14672.

*Psychrophrynella glauca* (4 specimens): PERU: Puno: Thiuni, Ollachea, CORBIDI 18729 (holotype), CORBIDI 18730, 16322, 16323 (paratopotypes).

*Psychrophrynella usurpator* (78 specimens): PERU: Cusco: Provincia Paucartambo, Kosñipata, MUSM 20011, 20873–81, 20896–20913, 20925–33, 20946–47, 20955–57, 21012–18, 26272–73, 26278–79, 26308, 27592, 27906, 27950, 28033–28047, 30303, 30305, 30396–30400, 30405–30409, 30471–30474.
