# Supplementary material for: Noblella thiuni sp. n., a new (singleton) species of minute terrestrial-breeding frog (Amphibia, Anura, Strabomantidae) from the montane forest of the Amazonian Andes of Puno, Peru
Source: PeerJ. 2019 Apr 23;7:e6780. doi: 10.7717/peerj.6780 (PMC6485238; doi:10.7717/peerj.6780)
Supplement: Appendix S2 — Genbank accession numbers for the taxa and genes sampled in this study.Collections abbreviations: AC –Alessandro Catenazzi Field Series; CFBHT –Celio F. B. Haddad Field Series; CORBIDI –Centro para Ornitología y Biodiversidad, Lima; CBF –Colección Boliviana de Fauna; KU –Kansas University, Lawrence; MNCN –Museo Nacional de Ciencias Naturales, Madrid; MUBI –Museo de Biodiversidad del Perú, Cusco; MUSM –Museo de Historia Natural de la Universidad de San Marcos, Lima. [file peerj-07-6780-s002.docx]

| **Taxon** | **Voucher Nr.** | **16S** |
| --- | --- | --- |
| *Holoaden luederwaldti* | CFBHT07810 | KU495249 |
| *Barycholos ternetzi* | CFBHT04408 | KU495152 |
| *Bryophryne bakersfield* | MUBI6022 | MF186341 |
| *Bryophryne cophites* | AC270.07 | KY652641 |
| *Bryophryne hanssaueri* | MUSM27567 | KY652642 |
| *Bryophryne nubilosus* | MUSM27882 | KY652643 |
| *Bryophryne phuyuhampatu* | CORBIDI18226 | MF419256 |
| *Microkayla chilina* | MNCN43774 | MF186416 |
| *Microkayla iatamasi* | MNCN42054 | MF186368 |
| *Microkayla katantika* | CBF6012 | MF186380 |
| *Noblella lochites* | KU177356 | EU186699 |
| *Noblella madreselva* | CORBIDI15770 | TBE |
| *Noblella myrmecoides* | CORBIDI PV45 | TBE |
| *Noblella pygmaea* | MUSM24536 | KY652645 |
| *Noblella* sp. R | MUSM27582 | KY652646 |
| *Noblella* sp. SP | AC58.18 | TBE |
| *Noblella thiuni* sp. n. | CORBIDI18723 | MK072732 |
| *Psychrophrynella chirihampatu* | MUBI14664 | KU884560 |
| *Psychrophrynella glauca* | CORBIDI18729 | MG837565 |
| *Psychrophrynella* sp. P | AC116.09 | KY65266 |
| *Psychrophrynella* sp. R | AC148.07 | KY652661 |
| *Psychrophrynella usurpator* | AC186.09 | KY652662 |
|  |  |  |
|  |  |  |
|  |  |  |
|  |  |  |

**Appendix 2. Genbank accession numbers for the taxa and genes sampled in this study.** Collections abbreviations: AC – Alessandro Catenazzi Field Series; CFBHT – Celio F. B. Haddad Field Series; CORBIDI –Centro para Ornitología y Biodiversidad, Lima; CBF – Colección Boliviana de Fauna; KU – Kansas University, Lawrence; MNCN – Museo Nacional de Ciencias Naturales, Madrid; MUBI – Museo de Biodiversidad del Perú, Cusco; MUSM – Museo de Historia Natural de la Universidad de San Marcos, Lima.
